# Brief review of image denoising techniques

**DOI:** 10.1186/s42492-019-0016-7

**Published:** 2019-07-08

**Authors:** Linwei Fan, Fan Zhang, Hui Fan, Caiming Zhang

**Affiliations:** 1 School of Software, Shandong University, ShunHua Road No.1500, Jinan, 250101 China; 2 Shandong Co-Innovation Center of Future Intelligent Computing, BinHai Road No.191, Yantai, 264005 China; 3 Shandong Province Key Lab of Digital Media Technology, Shandong University of Finance and Economics, Bicyclic East Road No. 7366, Jinan, 250061 China

**Keywords:** Image denoising, Non-local means, Sparse representation, Low-rank, Convolutional neural network

## Abstract

With the explosion in the number of digital images taken every day, the demand for more accurate and visually pleasing images is increasing. However, the images captured by modern cameras are inevitably degraded by noise, which leads to deteriorated visual image quality. Therefore, work is required to reduce noise without losing image features (edges, corners, and other sharp structures). So far, researchers have already proposed various methods for decreasing noise. Each method has its own advantages and disadvantages. In this paper, we summarize some important research in the field of image denoising. First, we give the formulation of the image denoising problem, and then we present several image denoising techniques. In addition, we discuss the characteristics of these techniques. Finally, we provide several promising directions for future research.

## Introduction

Owing to the influence of environment, transmission channel, and other factors, images are inevitably contaminated by noise during acquisition, compression, and transmission, leading to distortion and loss of image information. With the presence of noise, possible subsequent image processing tasks, such as video processing, image analysis, and tracking, are adversely affected. Therefore, image denoising plays an important role in modern image processing systems.

Image denoising is to remove noise from a noisy image, so as to restore the true image. However, since noise, edge, and texture are high frequency components, it is difficult to distinguish them in the process of denoising and the denoised images could inevitably lose some details. Overall, recovering meaningful information from noisy images in the process of noise removal to obtain high quality images is an important problem nowadays.

In fact, image denoising is a classic problem and has been studied for a long time. However, it remains a challenging and open task. The main reason for this is that from a mathematical perspective, image denoising is an inverse problem and its solution is not unique. In recent decades, great achievements have been made in the area of image denoising [1–4], and they are reviewed in the following sections.

The remainder of this paper is organized as follows. In Section “Image denoising problem statement”, we give the formulation of the image denoising problem. Sections “Classical denoising method, Transform techniques in image denoising, CNN-based denoising methods” summarize the denoising techniques proposed up to now. Section “Experiments” presents extensive experiments and discussion. Conclusions and some possible directions for future study are presented in Section “Conclusions”.

## Image denoising problem statement

Mathematically, the problem of image denoising can be modeled as follows:

1 $$ y=x+n $$

where *y* is the observed noisy image, *x* is the unknown clean image, and *n* represents additive white Gaussian noise (AWGN) with standard deviation *σ*_*n*_, which can be estimated in practical applications by various methods, such as median absolute deviation [5], block-based estimation [6], and principle component analysis (PCA)-based methods [7]. The purpose of noise reduction is to decrease the noise in natural images while minimizing the loss of original features and improving the signal-to-noise ratio (SNR). The major challenges for image denoising are as follows:

- flat areas should be smooth,
- edges should be protected without blurring,
- textures should be preserved, and
- new artifacts should not be generated.

Owing to solve the clean image *x* from the Eq. (1) is an ill-posed problem, we cannot get the unique solution from the image model with noise. To obtain a good estimation image $\hat{x}$, image denoising has been well-studied in the field of image processing over the past several years. Generally, image denoising methods can be roughly classified as [3]: spatial domain methods, transform domain methods, which are introduced in more detail in the next couple of sections.

## Classical denoising method

Spatial domain methods aim to remove noise by calculating the gray value of each pixel based on the correlation between pixels/image patches in the original image [8]. In general, spatial domain methods can be divided into two categories: spatial domain filtering and variational denoising methods.

### Spatial domain filtering

Since filtering is a major means of image processing, a large number of spatial filters have been applied to image denoising [9–19], which can be further classified into two types: linear filters and non-linear filters.

Originally, linear filters were adopted to remove noise in the spatial domain, but they fail to preserve image textures. Mean filtering [14] has been adopted for Gaussian noise reduction, however, it can over-smooth images with high noise [15]. To overcome this disadvantage, Wiener filtering [16, 17] has further been employed, but it also can easily blur sharp edges. By using non-linear filters, such as median filtering [14, 18] and weighted median filtering [19], noise can be suppressed without any identification. As a non-linear, edge-preserving, and noise-reducing smoothing filter, Bilateral filtering [10] is widely used for image denoising. The intensity value of each pixel is replaced with a weighted average of intensity values from nearby pixels. One issue concerning the bilateral filter is its efficiency. The brute-force implementation takes O(N*r*^2^) time, which is prohibitively high when the kernel radius *r* is large.

Spatial filters make use of low pass filtering on pixel groups with the statement that the noise occupies a higher region of the frequency spectrum. Normally, spatial filters eliminate noise to a reasonable extent but at the cost of image blurring, which in turn loses sharp edges.

### Variational denoising methods

Existing denoising methods use image priors and minimize an energy function E to calculate the denoised image $\hat{x}$. First, we obtain a function E from a noisy image *y*, and then a low number is corresponded to a noise-free image through a mapping procedure. Then, we can determine a denoised image $\hat{x}$ by minimizing E:

2 $$ \hat{x}\in \arg \underset{x}{\min}\mathrm{E}(x) $$

The motivation for variational denoising methods of Eq. (2) is maximum a posterior (MAP) probability estimate. From a Bayesian perspective, the MAP probability estimate of *x* is

3 $$ \hat{x}=\arg \underset{x}{\max}\mathrm{P}\left(x\left|y\right.\right)=\arg \underset{x}{\max}\frac{\mathrm{P}\left(y\left|x\right.\right)\mathrm{P}(x)}{\mathrm{P}(y)} $$

which can be equivalently formulated as

4 $$ \hat{x}=\arg \underset{x}{\max}\log \mathrm{P}\left(y\left|x\right.\right)+\log \mathrm{P}(x) $$

where the first term P(*y|x*) is a likelihood function of *x*, and the second term P(*x*) represents the image prior. In the case of AWGN, the objective function can generally be formulated as

5 $$ \hat{x}=\arg \underset{x}{\min}\frac{1}{2}{\left\Vert y-x\right\Vert}_2^2+\lambda \mathrm{R}(x) $$

where ${\left\Vert y-x\right\Vert}_2^2$ is a data fidelity term that denotes the difference between the original and noisy images. R(x) =  ‐ logP(x) denotes a regularization term and λ is the regularization parameter. For the variational denoising methods, the key is to find a suitable image prior (R(*x*)). Successful prior models include gradient priors, non-local self-similarity (NSS) priors, sparse priors, and low-rank priors.

In the remainder of this subsection, several popular variational denoising methods are summarized.

#### Total variation regularization

Starting with Tikhonov regularization [20, 21], the advantages of non-quadratic regularizations have been explored for a long time. Although the Tikohonov method [20, 21] is the simplest one in which R(*x*) is minimized with the L2 norm, it over-smooths image details [22, 23]. To solve this problem, anisotropic diffusion-based [24, 25] methods have been used to preserve image details, nevertheless, the edges are still blurred [26, 27].

Meanwhile, to solve the issue of smoothness, total variation (TV)-based regularization [28] has been proposed. This is the most influential research in the field of image denoising. TV regularization is based on the statistical fact that natural images are locally smooth and the pixel intensity gradually varies in most regions. It is defined as follows [28]:

6 $$ {\mathrm{R}}_{\mathrm{TV}}(x)={\left\Vert \nabla x\right\Vert}_1 $$

where ∇*x* is the gradient of *x*.

It has achieved great success in image denoising because it can not only effectively calculate the optimal solution but also retain sharp edges. However, it has three major drawbacks: textures tend to be over-smoothed, flat areas are approximated by a piecewise constant surface resulting in a stair-casing effect and the image suffers from losses of contrast [29–32].

To improve the performance of the TV-based regularization model, extensive studies have been conducted in image smoothing by adopting partial differential equations [33–36]. For example, Beck et al. [36] proposed a fast gradient-based method for constrained TV, which is a general framework for covering other types of non-smooth regularizers. Although it improves the peak signal-to-noise rate (PSNR) values, it only accounts for the local characteristics of the image.

#### Non-local regularization

While local denoising methods have low time complexities, the performances of these methods are limited when the noise level is high. The reason for this is that the correlations of neighborhood pixels are seriously disturbed by high level noise. Lately, some methods have applied the NSS prior [37]. This is because images contain extensive similar patches at different locations. A pioneering work on non-local means (NLM) [38] used the weighted filtering of the NSS prior to achieve image denoising, which is the most notable improvement for the problem of image denoising. Its basic idea is to build a pointwise estimation of the image, where each pixel is obtained as a weighted average of pixels centered at regions that are similar to the region centered at the estimated pixel. For a given pixel *x*_*i*_ in an image *x*, NLM(*x*_*i*_) indicates the NLM-filtered value. Let ***x***_*i*_ and ***x***_*j*_ be image patches centered at *x*_*i*_ and *x*_*j*_, respectively. Let *w*_*i*, *j*_ be the weight of ***x***_*j*_ to ***x***_*i*_, which is computed by.

7 $$ {w}_{i,j}=\frac{1}{c_i}\exp \left(-\frac{{\left\Vert {\mathtt{x}}_i-{\mathtt{x}}_j\right\Vert}_2^2}{h}\right) $$

where *c*_*i*_ denotes a normalization factor, and *h* indicates a filter parameter. Different from local denoising methods, NLM can make full use of the information provided by the given images, which can be robust to noise. Since then, many improved versions have been proposed. Some studies focus on the acceleration of the algorithm [39–44], while others focus on how to enhance the performance of the algorithm [45–47].

By considering the first step of NLM [38] (the estimation of pixel similarities), regularization methods have been developed [48]. According to Eq. (5), the NSS prior is defined as [49].

8 $$ {\mathrm{R}}_{\mathrm{NSS}}(x)=\sum \limits_{x_i\in x}{\left\Vert {x}_i-\mathrm{NLM}\left({x}_i\right)\right\Vert}_2^2=\sum \limits_{x_i\in x}{\left\Vert {x}_i-{\boldsymbol{w}}_i^{\mathrm{T}}{\boldsymbol{\kappa}}_i\right\Vert}_2^2 $$

where **κ**_*i*_ and **w**_*i*_ denote column vectors; the former contains the central pixels around *x*_*i*_, and the latter contains all corresponding weights *w*_*i*, *j*_.

At present, most research on image denoising has shifted from local methods to non-local methods [50–55]. For instance, extensions of non-local methods to TV regularization have been proposed in refs. [37, 56]. Considering the respective merits of the TV and NLM methods, an adaptive regularization of NLM (R-NL) [56] has been proposed to combine NLM with TV regularization. The results showed that the combination of these two models was successful in removing noise. Nevertheless, structural information is not well preserved by these methods, which degrades the visual image quality. Moreover, further prominent extensions and improvements of NSS methods are based on learning the likelihood of image patches [57] and exploiting the low-rank property using weighted nuclear norm minimization (WNNM) [58, 59].

#### Sparse representation

Sparse representation merely requires that each image patch can be represented as a linear combination of several patches from an over-complete dictionary [12, 60]. Many current image denoising methods exploit the sparsity prior of natural images.

Sparse representation-based methods encode an image over an over-complete dictionary **D** with L1-norm sparsity regularization on the coding vector, i.e., $\underset{\boldsymbol{\upalpha}}{\min }{\left\Vert \boldsymbol{\upalpha} \right\Vert}_1\ s.t.x=\mathbf{D}\boldsymbol{\upalpha }$, resulting in a general model:

9 $$ \hat{\boldsymbol{\alpha}}=\arg\ \underset{\boldsymbol{\alpha}}{\min }{\left\Vert y-\boldsymbol{D}\boldsymbol{\alpha } \right\Vert}_2^2+\lambda {\left\Vert \boldsymbol{\alpha} \right\Vert}_1 $$

where **α** is a matrix containing vectors of sparse coefficients. Eq. (9) turns the estimation of *x* in Eq. (5) into **α**.

As a dictionary learning method, the sparse representation model can be learned from a dataset, as well as from the image itself with the K-singular value decomposition (K-SVD) algorithm [61, 62]. The basic idea behind K-SVD denoising is to learn the dictionary **D** from a noisy image *y* by solving the following joint optimization problem:

10 $$ \arg\ \underset{x,\boldsymbol{D},\boldsymbol{\alpha}}{\min}\lambda {\left\Vert y-x\right\Vert}_2^2+\sum \limits_i{\left\Vert {\boldsymbol{R}}_ix-\boldsymbol{D}{\alpha}_i\right\Vert}_2^2+\sum \limits_i{\mu}_i{\left\Vert {\alpha}_i\right\Vert}_1 $$

where **R**_*i*_ is the matrix extracting patch ***x***_*i*_ from image *x* at location *i*.

Since the learned dictionaries can more flexibly represent the image structures [63], sparse representation models with learned dictionaries perform better than designed dictionaries. As shown in ref. [61], the K-SVD dictionary achieves up to 1–2 dB better for bit rates less than 1.5 bits per pixel (where the sparsity model holds true) compared to all other dictionaries. However, methods in this category are all local, meaning they ignore the correlation between non-local information of the image. In the case of high noise, local information is seriously disturbed, and the result of denoising is not effective.

Coupled with the NSS prior [37], the sparsity from self-similarity properties of natural images, which has received significant attention in the image processing community, is widely applied for image denoising [64–66]. One representative work is the non-local centralized sparse representation (NCSR) model [66].

11 $$ {\boldsymbol{\alpha}}_y=\arg \underset{\boldsymbol{\alpha}}{\min }{\left\Vert y-\boldsymbol{D}\boldsymbol{\alpha } \right\Vert}_2^2+\lambda \sum \limits_{i=1}^N{\left\Vert {\boldsymbol{\alpha}}_i-{\boldsymbol{\beta}}_i\right\Vert}_1 $$

where **β**_*i*_ is a good estimation of **α**. Then, for each image patch ***x***_*i*_, **β**_*i*_ can be computed as the weighted average of **α**_*i*, *q*_:

12 $$ {\boldsymbol{\beta}}_i=\sum \limits_{q\in {S}_i}{w}_{i,q}{\boldsymbol{\alpha}}_{i,q} $$

where *w*_*i*, *q*_ $=\frac{1}{c_i}\exp \left(-\frac{{\left\Vert {\hat{\mathtt{x}}}_i-{\hat{\mathtt{x}}}_{i,q}\right\Vert}_2^2}{h}\right)$, ${\hat{\boldsymbol{x}}}_i$ is the estimation of ***x***_*i*_, and ${\hat{\boldsymbol{x}}}_{i,q}$ are the non-local similar patches to ${\hat{\boldsymbol{x}}}_i$ in a search window S_*i*_.

The NCSR model naturally integrates NSS into the sparse representation framework, and it is one of the most commonly considered image denoising methods at present. As mentioned in ref. [66], NCSR is very effective in reconstructing both smooth and textured regions. Despite the successful combination of the above two techniques, the iterative dictionary learning and non-local estimates of unknown sparse coefficients make this algorithm computationally demanding, which largely limits its applicability in many applications.

#### Low-rank minimization

Different from the sparse representation model, this low-rank-based model formats similar patches as a matrix. Each column of this matrix is a stretched patch vector. By exploiting the low-rank prior of the matrix, this model can effectively reduce the noise in an image [67, 68]. The low-rank method first appeared in the field of matrix filling, and it has made great progress under the drive of Cand $\overset{`}{\mathrm{e}}$ s and Ma [69]. In recent years, the low-rank model has achieved good denoising results, resulting in low-rank denoising methods being studied more often.

Low-rank approaches for the reconstruction of noisy data can be grouped in two categories: methods based on low rank matrix factorization (refs. [70–78]) and those based on nuclear norm minimization (NNM, ref. [58, 59, 79, 80]).

Methods in the first category typically approximate a given data matrix as a product of two matrices of fixed low rank. For example, in refs. [70, 71], a video denoising algorithm based on low-rank matrix recovery was proposed. In these methods, similar patches are decomposed by low-rank decomposition to remove noise from videos. Ref. [72] proposed an image denoising algorithm based on low-rank matrix recovery and obtained good results. In ref. [73], a hybrid noise removal algorithm based on low-rank matrix recovery was proposed. Dong et al. [74] proposed a low-rank method based on SVD to model the sparse representation of non-locally similar image patches. In this method, singular value iteration contraction in the BayesShrink framework was used to remove noise. The main limitation of these methods is that the rank must be provided as input, and values that are too low or too high will result in the loss of details or the preservation of noise, respectively.

Low-rank minimization is a non-convex non-deterministic polynomial (NP) hard problem [63]. Alternatively, methods based on NNM aim to find the lowest rank approximation **X** of an observed matrix **Y**. Let **Y** be a matrix of noisy patches. From **Y**, the low-rank matrix **X** can be estimated by the following NNM problem [80]:

13 $$ \hat{\boldsymbol{X}}=\arg\ \underset{\boldsymbol{X}}{\min }{\left\Vert \boldsymbol{Y}-\boldsymbol{X}\right\Vert}_F^2+\lambda {\left\Vert \boldsymbol{X}\right\Vert}_{\ast } $$

where ${\left\Vert \cdot \right\Vert}_F^2$ denotes the Frobenius norm, and the nuclear norm ${\left\Vert \boldsymbol{X}\right\Vert}_{\ast }=\sum \limits_i{\left\Vert {\sigma}_i\left(\boldsymbol{X}\right)\right\Vert}_1$, where *σ*_*i*_(***X***) is the *i*-th singular value of **X**. A closed-form solution of Eq. (13) has been proposed in ref. [80] and is shown in Eq. (14)

14 $$ \hat{\boldsymbol{X}}={\boldsymbol{US}}_{\lambda}\left(\boldsymbol{\varSigma} \right){\boldsymbol{V}}^{\mathrm{T}} $$

where ***Y = UΣV***^T^ is the SVD of **Y** and ***S***_*λ*_(***Σ***) = max(***Σ*** − *λ****I***, 0) is the singular value thresholding operator. For NNM [80], the weights of each singular value are equal, and the same threshold is applied to each singular value, however different singular values have different levels of importance.

Hence, on the basis of the NNM, Gu et al. [58, 59] proposed a WNNM model, which can adaptively assign weights to singular values of different sizes and denoise them using a soft threshold method. Given a weight vector **w**, the weighted nuclear norm proximal problem consists of finding an approximation **X** of **Y** that minimizes the following cost function:

15 $$ \hat{\boldsymbol{X}}=\arg\ \underset{\boldsymbol{X}}{\min }{\left\Vert \boldsymbol{Y}-\boldsymbol{X}\right\Vert}_F^2+{\left\Vert \boldsymbol{X}\right\Vert}_{\boldsymbol{w},\ast } $$

where ${\left\Vert \boldsymbol{X}\right\Vert}_{\boldsymbol{w},\ast }=\sum \limits_i{\left\Vert {w}_i{\sigma}_i\left(\boldsymbol{X}\right)\right\Vert}_1$ is the weighted nuclear norm of **X.** Here, *w*_*i*_ denotes the weight assigned to singular value *σ*_*i*_(***X***). As shown in ref. [58], Eq. (15) has a unique global minimum when the weights satisfy 0 ≤ *w*_1_ ≤ ⋯ ≤ *w*_*n*_:

16 $$ \hat{\boldsymbol{X}}={\boldsymbol{US}}_{\boldsymbol{w}}\left(\boldsymbol{\varSigma} \right){\boldsymbol{V}}^{\mathrm{T}} $$

where ***S***_***w***_(***Σ***) = max(***Σ*** − *Diag*(***w***), 0).

From ref. [58], we know that WNNM achieves advanced denoising performance and is more robust to noise strength than other NNMs. Besides, the low-rank theory has been widely used in artificial intelligence, image processing, pattern recognition, computer vision, and other fields [63]. Although most low-rank minimization methods (especially the WNNM method) outperform previous denoising methods, the computational cost of the iterative boosting step is relatively high.

## Transform techniques in image denoising

Image denoising methods have gradually developed from the initial spatial domain methods to the present transform domain methods. Initially, transform domain methods were developed from the Fourier transform, but since then, a variety of transform domain methods gradually emerged, such as cosine transform, wavelet domain methods [81–83], and block-matching and 3D filtering (BM3D) [55]. Transform domain methods employ the following observation: the characteristics of image information and noise are different in the transform domain.

### Transform domain filtering methods

In contrast with spatial domain filtering methods, transform domain filtering methods first transform the given noisy image to another domain, and then they apply a denoising procedure on the transformed image according to the different characteristics of the image and its noise (larger coefficients denote the high frequency part, i.e., the details or edges of the image, smaller coefficients denote the noise). The transform domain filtering methods can be subdivided according to the chosen basis transform functions, which may be data adaptive or non-data adaptive [84].

#### Data adaptive transform

Independent component analysis (ICA) [85, 86] and PCA [65, 87] functions are adopted as the transform tools on the given noisy images. Among them, the ICA method has been successfully implemented for denoising non-Gaussian data. These two kinds of methods are data adaptive, and the assumptions on the difference between the image and noise still hold. However, their main drawback is high-computational cost because they use sliding windows and require a sample of noise-free data or at least two image frames from the same scene. However, in some applications, it might be difficult to obtain noise-free training data.

#### Non-data adaptive transform

The non-data adaptive transform domain filtering methods can be further subdivided into two domains, namely spatial-frequency domain and wavelet domain.

Spatial-frequency domain filtering methods use low pass filtering by designing a frequency domain filter that passes all frequencies lower than and attenuates all frequencies higher than a cut-off frequency [14, 16]. In general, after being transformed by low-pass filters, such as Fourier transform, image information mainly spreads in the low frequency domain, while noise spreads in the high frequency domain. Thus, we can remove noise by selecting specific transform domain features and transforming them back to the image domain [88]. Nevertheless, these methods are time-consuming and depend on the cut-off frequency and filter function behavior.

As the most investigated transform in denoising, the wavelet transform [89] decomposes the input data into a scale-space representation. It has been proved that wavelets can successfully remove noise while preserving the image characteristics, regardless of its frequency content [90–95]. Similar to spatial domain filtering, filtering operations in the wavelet domain can also be subdivided into linear and non-linear methods. Since the wavelet transform has many good characteristics, such as sparseness and multi-scale, it is still an active area of research in image denoising [96]. However, the wavelet transform heavily relies on the selection of wavelet bases. If the selection is inappropriate, image shown in the wavelet domain cannot be well represented, which causes poor denoising effect. Therefore, this method is not adaptive.

### BM3D

As an effective and powerful extension of the NLM approach, BM3D, which was proposed by Dabov et al. [55], is the most popular denoising method. BM3D is a two-stage non-locally collaborative filtering method in the transform domain. In this method, similar patches are stacked into 3D groups by block matching, and the 3D groups are transformed into the wavelet domain. Then, hard thresholding or Wiener filtering with coefficients is employed in the wavelet domain. Finally, after an inverse transform of coefficients, all estimated patches are aggregated to reconstruct the whole image. However, when the noise increases gradually, the denoising performance of BM3D decreases greatly and artifacts are introduced, especially in flat areas.

To improve denoising performance, many improved versions of BM3D have appeared [97, 98]. For example, Maggioni et al. [98] recently proposed the block-matching and 4D filtering (BM4D) method, which is an extension of BM3D to volumetric data. It utilizes cubes of voxels, which are stacked into a 4-D group. The 4-D transform applied on the group simultaneously exploits the local correlation and non-local correlation of voxels. Thus, the spectrum of the group is highly sparse, leading to very effective separation of signal and noise through coefficient shrinkage.

## CNN-based denoising methods

In general, the solving methods of the objective function in Eq. (7) build upon the image degradation process and the image priors, and it can be divided into two main categories: model-based optimization methods and convolutional neural network (CNN)-based methods. The variational denoising methods discussed above belong to model-based optimization schemes, which find optimal solutions to reconstruct the denoised image. However, such methods usually involve time-consuming iterative inference. On the contrary, the CNN-based denoising methods attempt to learn a mapping function by optimizing a loss function on a training set that contains degraded-clean image pairs [99, 100].

Recently, CNN-based methods have been developed rapidly and have performed well in many low-level computer vision tasks [101, 102]. The use of a CNN for image denoising can be tracked back to [103], where a five-layer network was developed. In recent years, many CNN-based denoising methods have been proposed [99, 104–108]. Compared to that of ref. [103], the performance of these methods has been greatly improved. Furthermore, CNN-based denoising methods can be divided into two categories: multi-layer perception (MLP) models and deep learning methods.

### MLP models

MLP-based image denoising models include auto-encoders proposed by Vincent et al. [104] and Xie et al. [105]. Chen et al. [99] proposed a feed-forward deep network called the trainable non-linear reaction diffusion (TNRD) model, which achieved a better denoising effect. This category of methods has several advantages. First, these methods work efficiently owing to fewer ratiocination steps. Moreover, because optimization algorithms [77] have the ability to derive the discriminative architecture, these methods have better interpretability. Nevertheless, interpretability can increase the cost of performance; for example, the MAP model [106] restricts the learned priors and inference procedure.

### Deep learning-based denoising methods

The state-of-the-art deep learning denoising methods are typically based on CNNs. The general model for deep learning-based denoising methods is formulated as

17 $$ \underset{\Theta}{\min } loss\left(\hat{x},x\right),s.t.\hat{x}=F\left(y,\sigma; \Theta \right) $$

where *F*(⋅) denotes a CNN with parameter set Θ, and *loss*(⋅) denotes the loss function. *loss*(⋅) is used to estimate the proximity between the denoised image $\hat{x}$ and the ground-truth *x*. Owing to their outstanding denoising ability, considerable attention has been focused on deep learning-based denoising methods.

Zhang et al. [106] introduced residual learning and batch standardization into image denoising for the first time; they also proposed feed-forward denoising CNNs (DnCNNs). The aim of the DnCNN model is to learn a function $\hat{x}=F\left(y;{\Theta}_{\sigma}\right)$ that maps between *y* and $\hat{x}$. The parameters Θ_*σ*_ are trained for noisy images under a fixed variance σ. There are two main characteristics of DnCNNs: the model applies a residual learning formulation to learn a mapping function, and it combines it with batch normalization to accelerate the training procedure while improving the denoising results. Specifically, it turns out that residual learning and batch normalization can benefit each other, and their integration is effective in speeding up the training and boosting denoising performance. Although a trained DnCNN can also handle compression and interpolation errors, the trained model under σ is not suitable for other noise variances.

When the noise level σ is unknown, the denoising method should enable the user to adaptively make a trade-off between noise suppression and texture protection. The fast and flexible denoising convolutional neural network (FFDNet) [107] was introduced to satisfy these desirable characteristics. In particular, FFDNet can be modeled as $\hat{x}=F\left(y,\mathrm{M};\Theta \right)$ (M denotes a noise level map), which is a main contribution. For FFDNet, M indicates an input while the parameter set Θ are fixed for noise level. Another major contribution is that FFDNet acts on down-sampled sub-images, which speeds up the training and testing and also expands the receptive field. Thus, FFDNet is quite flexible to different noises.

Although this method is effective and has a short running time, the time complexity of the learning process is very high. The development of CNN-based denoising methods has enhanced the learning of high-level features by using a hierarchical network.

## Experiments

For a comparative study, the existing denoising methods adopt two factors (visual analysis and performance metrics) to analyze the denoising performance.

Currently, we cannot find any mathematical or specific methods to evaluate the visual analysis. In general, there are three criteria for visual analysis: (1) significant degree of artifacts, (2) protection of edges, and (3) reservation of textures. For image denoising methods, several performance metrics are adopted to evaluate accuracy, e.g., PSNR and structure similarity index measurement (SSIM) [109].

**Fig. 1:**
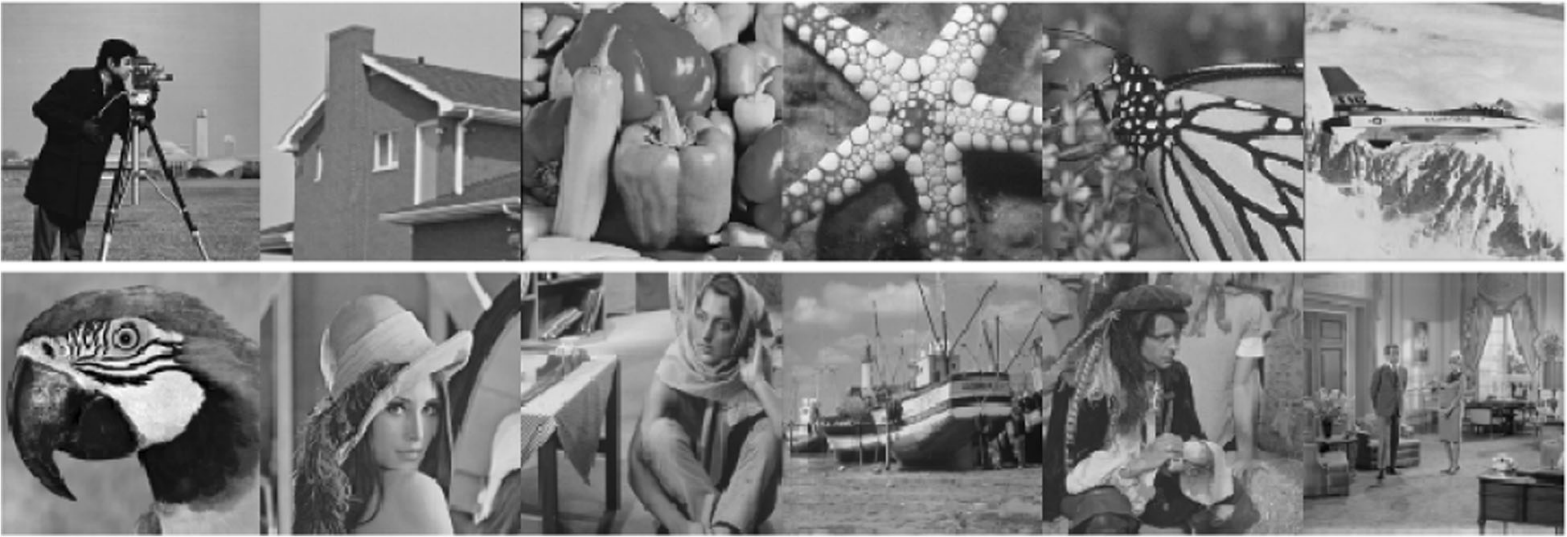
Twelve test images from Set12 dataset

In this study, all image denoising methods work on noisy images under three different noise variances *σ* ∈ [30, 50, 75]. For the test images, we use two datasets for a thorough evaluation: BSD68 [110] and Set12. The BSD68 dataset consists of 68 images from the separate test set of the BSD dataset. The Set12 dataset, which is shown in Fig. 1, is a collection of widely used testing images. The sizes of the first seven images are 256 × 256, and the sizes of the last five images are 512 × 512.

### Metrics of denoising performance

To evaluate the performance metrics of image denoising methods, PSNR and SSIM [109] are used as representative quantitative measurements:

Given a ground truth image *x*, the PSNR of a denoised image $\hat{x}$ is defined by

18 $$ PSNR\left(x,\hat{x}\right)=10\cdot {\log}_{10}\left(\frac{255^2}{{\left\Vert x-\hat{x}\right\Vert}_2^2}\right) $$

In addition, the SSIM index is calculated by

19 $$ SSIM\left(x,\hat{x}\right)=\frac{\left(2{\mu}_x{\mu}_{\hat{x}}+{C}_1\right)\left(2{\sigma}_{x\hat{x}}+{C}_2\right)}{\left({\mu}_x^2+{\mu}_{\hat{x}}^2+{C}_1\right)\left({\sigma}_x^2+{\sigma}_{\hat{x}}^2+{C}_2\right)} $$

where ${\mu}_x,{\mu}_{\hat{x}},{\sigma}_x$, and ${\sigma}_{\hat{x}}$ are the means and variances of *x* and $\hat{x}$, respectively, ${\sigma}_{x\hat{x}}$ is the covariance between *x* and $\hat{x}$, and C_1_ and C_2_ are constant values used to avoid instability. While quantitative measurements cannot reflect the visual quality perfectly, visual quality comparisons on a set of images are necessary. Besides the noise removal effect, edge and texture preservation is vital for evaluating a denoising method.

### Comparison methods

A comprehensive evaluation is conducted on several state-of-the-art methods, including Wiener filtering [16], Bilateral filtering [10], PCA method [87], Wavelet transform method [89], BM3D [55], TV-based regularization [28], NLM [38], R-NL [56], NCSR model [66], LRA_SVD [78], WNNM [58], DnCNN [106], and FFDNet [107]. Among them, the first five are all filtering methods, while the last two are CNN-based methods. The remaining algorithms are variational denoising methods.

In our experiments, the code and implementations provided by the original authors are used. All the source codes are run on an Intel Core i5–4570 CPU 3.20 GHz with 16 GB memory. The core part of the BM3D calculation is implemented with a compiled C++ mex-function and is performed in parallel, while the other methods are all conducted using MATLAB.

### Comparison of filtering methods and variational denoising methods

**Fig. 2:**
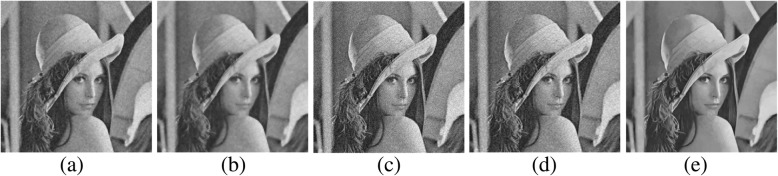
Visual comparisons of denoising results on Lena image corrupted by additive white Gaussian noise with standard deviation 30: **a** Wiener filtering [16] (PSNR = 27.81 dB; SSIM = 0.707); **b** Bilateral filtering [10] (PSNR = 27.88 dB; SSIM = 0.712); **c** PCA method [87] (PSNR = 26.68 dB; SSIM = 0.596); **d** Wavelet transform domain method [89] (PSNR = 21.74 dB; SSIM = 0.316); **e** Collaborative filtering: BM3D [55] (PSNR = 31.26 dB; SSIM = 0.845)

**Fig. 3:**
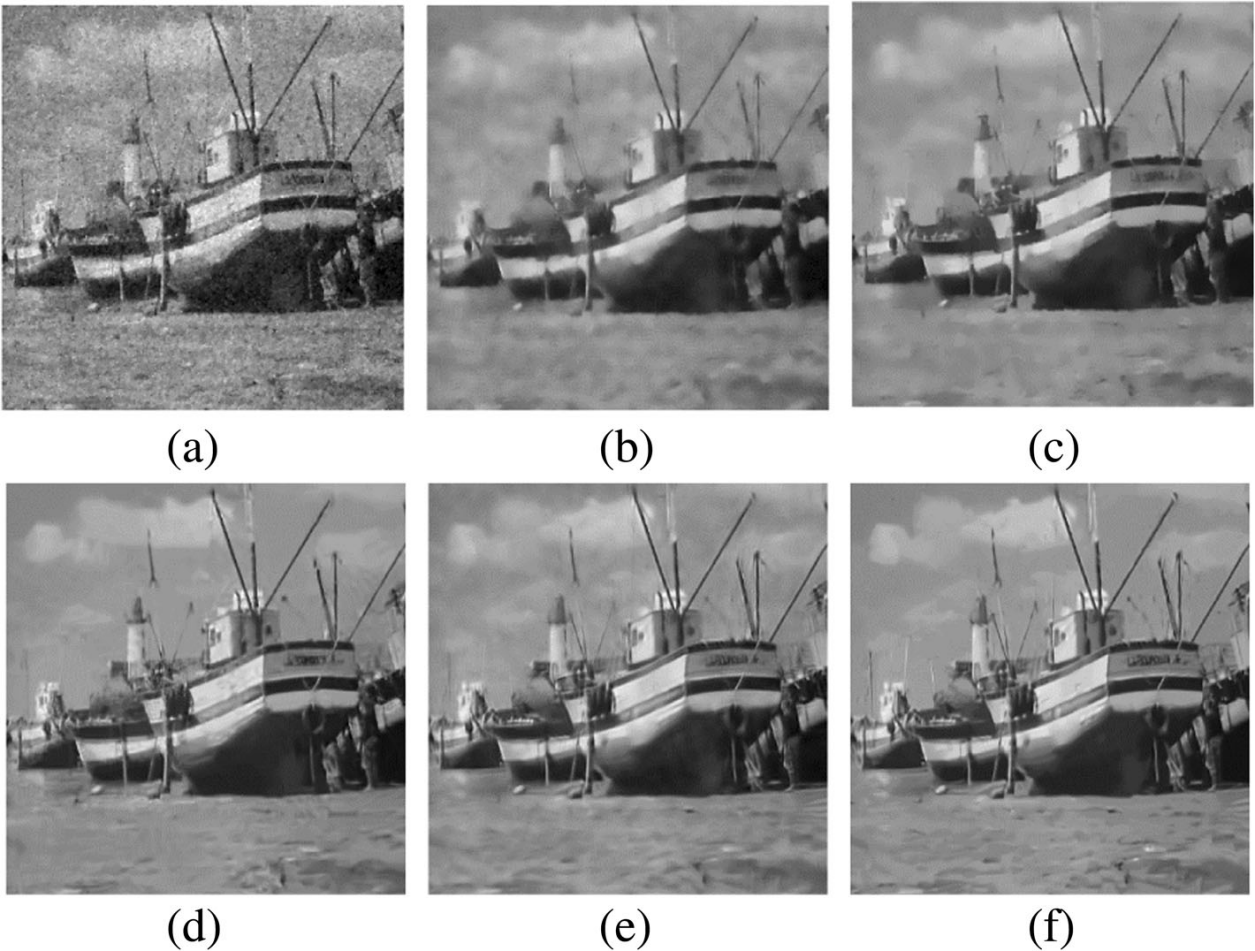
Visual comparisons of denoising results on Boat image corrupted by additive white Gaussian noise with standard deviation 50: **a** TV-based regularization [28] (PSNR = 22.95 dB; SSIM = 0.456); **b** NLM [38] (PSNR = 24.63 dB; SSIM = 0.589); **c** R-NL [56] (PSNR = 25.42 dB; SSIM = 0.647); **d** NCSR model [66] (PSNR = 26.48 dB; SSIM = 0.689); **e** LRA_SVD [78] (PSNR = 26.65 dB; SSIM = 0.684); **f** WNNM [58] (PSNR = 26.97 dB; SSIM = 0.708)

We first present experimental results of image denoising on the 12 test images from the Set12 dataset. Figures 2 and 3 show the denoising comparison results by the filtering methods variational denoising methods, respectively.

From Fig. 2, one can see that the spatial filters (Wiener filtering [16] and Bilateral filtering [10]) denoise the image better than the transform domain filtering methods (PCA method [87] and Wavelet transform domain method [89]). However, the spatial filters eliminate high frequency noise at the expense of blurring fine details and sharp edges. The result of collaborative filtering (BM3D) [55] has big potential for noise reduction and edge protection.

In Fig. 3, the visual evaluation shows that the denoising result of the TV-based regularization [28] smooths the textures and generates artifacts. Although the R-NL [56] and NLM [38] methods can obtain better performances, these two methods have difficulty restoring tiny structures. Meanwhile, we find that the representative low-rank-based methods (WNNM [58], LRA_SVD [78]) and the sparse coding scheme NCSR [66] produce better results in homogenous regions because the underlying clean patches share similar features, so they can be approximated by a low-rank or sparse coding problem.

### Comparison of CNN-based denoising methods

Here, we compare the denoising results of the CNN-based methods (DnCNN [106] and FFDNet [107]) with those of several current effective image denoising methods, including BM3D [55] and WNNM [58]. To the best of our knowledge, BM3D has been the most popular denoising method over recent years, and WNNM is a successful scheme that has been proposed recently.

Table 1 reports the PSNR results on the BSD68 dataset. From Table 1, the following observations can be made. First, FFDNet [107] outperforms BM3D [55] by a large margin and outperforms WNNM [58] by approximately 0.2 dB for a wide range of noise levels. Secondly, FFDNet is slightly inferior to DnCNN [106] when the noise level is low (e.g., σ ≤ 25), but it gradually outperforms DnCNN as the noise level increases (e.g., σ > 25).

**Table 1:** Average peak signal-to-noise ratio (dB) results for different methods on BSD68 with noise levels of 15, 25, 50 and 75

Methods	BM3D	WNNM	DnCNN	FFDNet
σ = 15	31.07	31.37	31.72	31.62
σ = 25	28.57	28.83	29.23	29.19
σ = 50	25.62	25.87	26.23	26.30
σ = 75	24.21	24.40	26.64	24.78

**Fig. 4:**
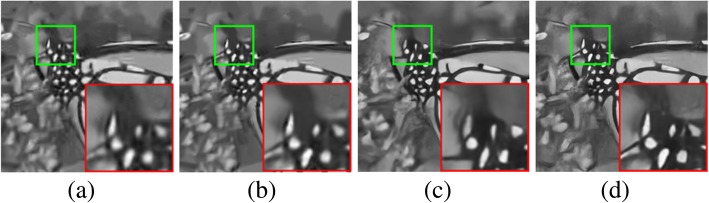
Visual comparisons of denoising results on Monarch image corrupted by additive white Gaussian noise with standard deviation 75: **a** BM3D [55] (PSNR = 23.91 dB); **b** WNNM [58] (PSNR = 24.31 dB); **c** DnCNN [106] (PSNR = 24.71 dB); **d** FFDNet [107] (PSNR = 24.99 dB)

In Fig. 4, we can see that the details of the antennas and contour areas are difficult to recover. BM3D [55] and WNNM [58] blur the fine textures, whereas the other two methods restore more textures. This is because Monarch has many repetitive structures, which can be effectively exploited by NSS. Moreover, the contour edges of these regions are much sharper and look more natural. Overall, FFDNet [107] produces the best perceptual quality of denoised images.

## Conclusions

As the complexity and requirements of image denoising have increased, research in this field is still in high demand. We have introduced the recent developments of several image denoising methods and discussed their merits and drawbacks in this paper. Recently, the rise of NLM has replaced the traditional local denoising model, which has created a new theoretical branch, leading to significant advances in image denoising methods, including sparse representation, low-rank, and CNN (more specifically deep learning)-based denoising methods. Although the image sparsity and low-rank priors have been widely used in recent years, CNN-based methods, which have been proved to be effective, have undergone rapid growth in this time.

Despite the many in-depth studies on removing AWGN, few have considered real image denoising. The major obstacle is the complexity of real noises because AWGN is much simpler than real noises. In this situation, the thorough evaluation of a denoiser is a difficult task. There are several components (e.g., white balance, color demosaicing, noise reduction, color transform, and compression) contained in the in-camera pipeline. The output image quality is affected by some external and internal conditions, such as illumination, CCD/CMOS sensors, and camera shaking.

Although deep learning is developing rapidly, it is not necessarily an effective way to solve the denoising problem. The main reason for this is that real-world denoising processes lack image pairs for training. To the best of our knowledge, the existing denoising methods are all trained by simulated noisy data generated by adding AWGN to clean images. Nevertheless, for the real-world denoising process, we find that the CNNs trained by such simulated data are not sufficiently effective.

In summary, this paper aims to offer an overview of the available denoising methods. Since different types of noise require different denoising methods, the analysis of noise can be useful in developing novel denoising schemes. For future work, we must first explore how to deal with other types of noise, especially those existing in real life. Secondly, training deep models without using image pairs is still an open problem. Besides, the methodology of image denoising can also be expanded to other applications [111, 112].

## Data Availability

The datasets used and/or analyzed during the current study are available from the corresponding author on reasonable request.

## Abbreviations

AWGN: Additive white Gaussian noise
BM3D: Block-matching and 3D filtering
CNN: Convolutional netural network
DnCNN: Feed-forward denoising convolutional neural network
FFDNet: Fast and flexible denoising convolutional neural network
ICA: Independent component analysis
K-SVD: K-singular value decomposition
MAP: Maximum a posterior
MLP: Multi-layer perception model
NCSR: Non-local centralized sparse representation
NLM: Non-local means
NNM: Nuclear norm minimization
NP: Non-deterministic polynomial
NSS: Non-local self-similarity
PCA: Principle component analysis
PSNR: Peak signal to noise rate
SNR: Signal-to-noise ratio
SSIM: Structure similarity index measurement
TV: Total variation
WNNM: Weighted nuclear norm minimization
